# Awake prone positioning for non-intubated patients with COVID-19-related acute hypoxic respiratory failure: a systematic review based on eight high-quality randomized controlled trials

**DOI:** 10.1186/s12879-023-08393-8

**Published:** 2023-06-19

**Authors:** Wen Cao, Nannan He, Yannian Luo, Zhiming Zhang

**Affiliations:** 1 Department of Critical Medicine, the Second Hospital of Lanzhou University, Lanzhou, 730030 China; 2 Department of Oncology, Gansu Provincial Hospital of Traditional Chinese Medicine, Lanzhou, 730030 China

**Keywords:** COVID-19, Acute hypoxic respiratory failure, Non-intubated, Awake prone positioning, Randomized controlled trials, Systematic review

## Abstract

**Background:**

Awake prone positioning has been widely used in non-intubated patients with acute hypoxic respiratory failure (AHRF) due to COVID-19, but the evidence is mostly from observational studies and low-quality randomized controlled trials (RCTs), with conflicting results from published studies. A systematic review of published high-quality RCTs to resolve the controversy over the efficacy and safety of awake prone positioning in non-intubated patients with AHRF due to COVID-19.

**Methods:**

Candidate studies were identified through searches of PubMed, Web of Science, Cochrane, Embase, Scopus databases from December 1, 2019 to November 1, 2022. Literature screening, data extraction and risk of bias assessment were independently conducted by two researchers.

**Results:**

Eight RCTs involving 2657 patients were included. Meta-analysis of fixed effects models showed that awake prone positioning did not increase mortality(OR = 0.88, 95%CI [0.72, 1.08]), length of stay in ICU (WMD = 1.14, 95%CI [-0.45, 2.72]), total length of stay (WMD = 0.11, 95%CI [-1.02, 1.23]), or incidence of adverse events (OR = 1.02, 95%CI [0.79, 1.31]) compared with usual care, but significantly reduced the intubation rate (OR = 0.72, 95%CI [0.60, 0.86]). Similar results were found in a subgroup analysis of patients who received only high flow nasal cannula (Mortality: OR = 0.86, 95%CI [0.70, 1.05]; Intubation rate: OR = 0.69, 95%CI [0.58, 0.83]). All eight RCTs had high quality of evidence, which ensured the reliability of the meta-analysis results.

**Conclusions:**

Awake prone positioning is safe and feasible in non-intubated patients with AHRF caused by COVID-19, and can significantly reduce the intubation rate. More studies are needed to explore standardized implementation strategies for the awake prone positioning.

**Trial registration:**

CRD42023394113.

**Supplementary Information:**

The online version contains supplementary material available at 10.1186/s12879-023-08393-8.

## Background

The ongoing global pandemic of COVID-19 has led to significant morbidity and mortality, and poses unique challenges to medical system, including severe shortages of medical staff, funding, ICU beds, and the number of mechanical ventilators [1]. While the majority of patients are asymptomatic or mildly infected, about 14% of patients develop more severe disease, mainly acute hypoxic respiratory failure (AHRF). AHRF is characterized by hypoxemia, increased respiratory rate, and respiratory distress [2–4]. Such patients should be admitted to the high dependency or intensive care unit (ICU) for treatment, but given the rapid increase in cases during the recent pandemic, many of these units and ICU have been overwhelmed in providing care [2, 5]. New ways to reduce or improve the severity of the disease are urgently needed. awake prone positioning is one of the potential measures.

Awake prone positioning mainly involves rotating the patient from supine to ventral position while awake and not intubated to allow for greater expansion of lung tissue in the dorsal area [6]. Prior to the pandemic, awake prone positioning had been used to reduce intubation rates and mortality in patients with AHRF and acute respiratory distress syndrome (ARDS) [6, 7]. Today, awake prone positioning has also achieved positive results in patients with hypoxic respiratory failure caused by COVID-19 (reduced intubation rate and improved oxygen saturation) [8, 9]. However, the current findings are controversial because some studies suggest that awake prone positioning does not reduce intubation rates and mortality in patients [10, 11]. In conclusion, there is a lack of high-quality evidence to prove whether awake prone positioning can be used in patients with hypoxic respiratory failure caused by COVID-19.

Although there have been several systematic reviews/Meta-analyses (SRs/MAs) exploring the effects of awake prone positioning on patients with AHRF caused by COVID-19, these studies have the following problems and inconsistent results. First, previous SRs/MAs combined data from different types of studies (observational studies and RCTs), resulting in high heterogeneity among different studies and reducing the reliability of meta-analysis results [12, 13]. Second, the quality of evidence in published retrospective and observational studies included in SRs/MAs is low and does not provide reliable evidence for clinical practice [12, 14]. In addition, the number of databases searched for published SRs/MAs is too small and the sources are not comprehensive, which may miss important research results or create publication bias [15]. More importantly, several high-quality RCTs published recently have provided new data [16, 17]. Therefore, it is necessary to update relevant evidence in time.

In this study, we aimed to conduct an updated meta-analysis to systematically explore the efficacy and safety of awake prone positioning in patients with AHRF caused by COVID-19, with a view to providing the latest and most reliable evidence for the treatment of patients.

## Methods

This systematic review and meta-analysis followed the guidelines of the Preferred Reporting Items for Systematic Reviews and Meta-Analyses (PRISMA) [18]. The protocol was registered (CRD42023394113) on PROSPERO (www.crd.york.ac.uk/prospero).

### Inclusion and exclusion criteria

#### Patients (P)

Non-intubated patients with AHRF caused by COVID-19. The diagnostic criteria of hypoxic respiratory failure are PaO2:FiO2 ratio ≤ 300 mmHg, or no specific diagnostic criteria have been reported in the study, but patients with hypoxic failure are clearly described.

#### Interventions (I)

The patient is in the awake prone positioning for at least 6 h a day and is in the awake prone positioning for as long as possible [19].

#### Control (C)

Patients receive only usual care and no restrictions on postures.

#### Outcome (O)

1) Primary outcomes: intubation rate, mortality. Mortality is defined as the number of deaths caused by COVID-19 during treatment divided by the total number of patients receiving treatment. 2) Secondary outcomes: hospital length of stay and incidence of adverse events.

### Type of study (S): RCTs

#### Exclusion criteria

1) Patients with AHRF due to bronchial asthma, heart failure or pulmonary embolism, or patients with COVID-19 who received endotracheal intubation, were excluded. 2) Observational studies and studies without a control group were excluded. 3) Excluded studies that did not report expected outcome indicators. 4) Editorials, narrative reviews, letters, and conference abstracts were excluded. 5) Studies without full text or primary data that could not be obtained were excluded.

### Data sources and searches

Candidate studies were identified through searches of PubMed, Web of Science, Cochrane, Embase, Scopus databases from December 1, 2019 to November 1, 2022. The following terms were combined to design the search strategy: (SARS-CoV-2 OR SARS-CoV2 OR ‘severe acute respiratory syndrome coronavirus 2’ OR 2019-nCoV OR 2019nCoV OR coronavirus OR covid-19 OR COVID19 OR COVID-19) AND (supine position OR dorsal position OR prone position OR lateral position OR ventilation position OR ventilatory position OR ventilation posture OR ventilatory posture). Further details of the search strategy are shown in Supplementary Table 1. Reference lists of included studies and of previously published guidelines and systematic reviews were also searched.

### Literature screening, data extraction and bias risk assessment

First, titles and abstracts were reviewed to identify studies that met the inclusion criteria. Second, full texts were obtained to determine the final required studies. After identifying the studies that ultimately met the inclusion criteria, we first contacted the corresponding author of the paper by email to obtain raw data. If there is no response, we will extract the required information from the paper according to the information extraction table made in advance, including, 1) Basic information: Author, year of publication, country, type of study, disease, oxygen supply pattern, location, sample size for trial and control groups, age, details of care. 2) Primary and secondary outcome indicators. 3) Key information for bias risk assessment. Finally, the Cochrane bias risk assessment tool was used to assess the risk of bias in included studies [20]. The above processes were carried out independently by two researchers (CW, HN) with rich experience in systematic review production, and any discrepancy was decided with the assistance of the third researcher (ZZ).

### Statistical analysis

Statistical analysis was performed by RevMan 5.4.1 software. Intubation rate, mortality and adverse events were measured by odds ratio (OR), and the length of stay in hospital was measured by weighted mean difference (WMD). All effects were expressed by 95% confidence interval (95% CI). When there was a large heterogeneity between the included studies (P < 0.05 and I^2^ ≥ 50%), subgroup analysis, sensitivity analysis and meta-regression were used to further analyze the sources of heterogeneity. When heterogeneity was found to exist even after all methods were taken to reduce heterogeneity, a random effects model was used to analyze the data. Conversely, when heterogeneity was not present, the fixed-effects model was used for data analysis. In addition, when less than 10 studies were included, it was difficult to judge whether there was publication bias according to the symmetry of the funnel plot, so Egger’s test was used to quantitatively detect publication bias.

## Results

### Basic information

**Fig. 1:**
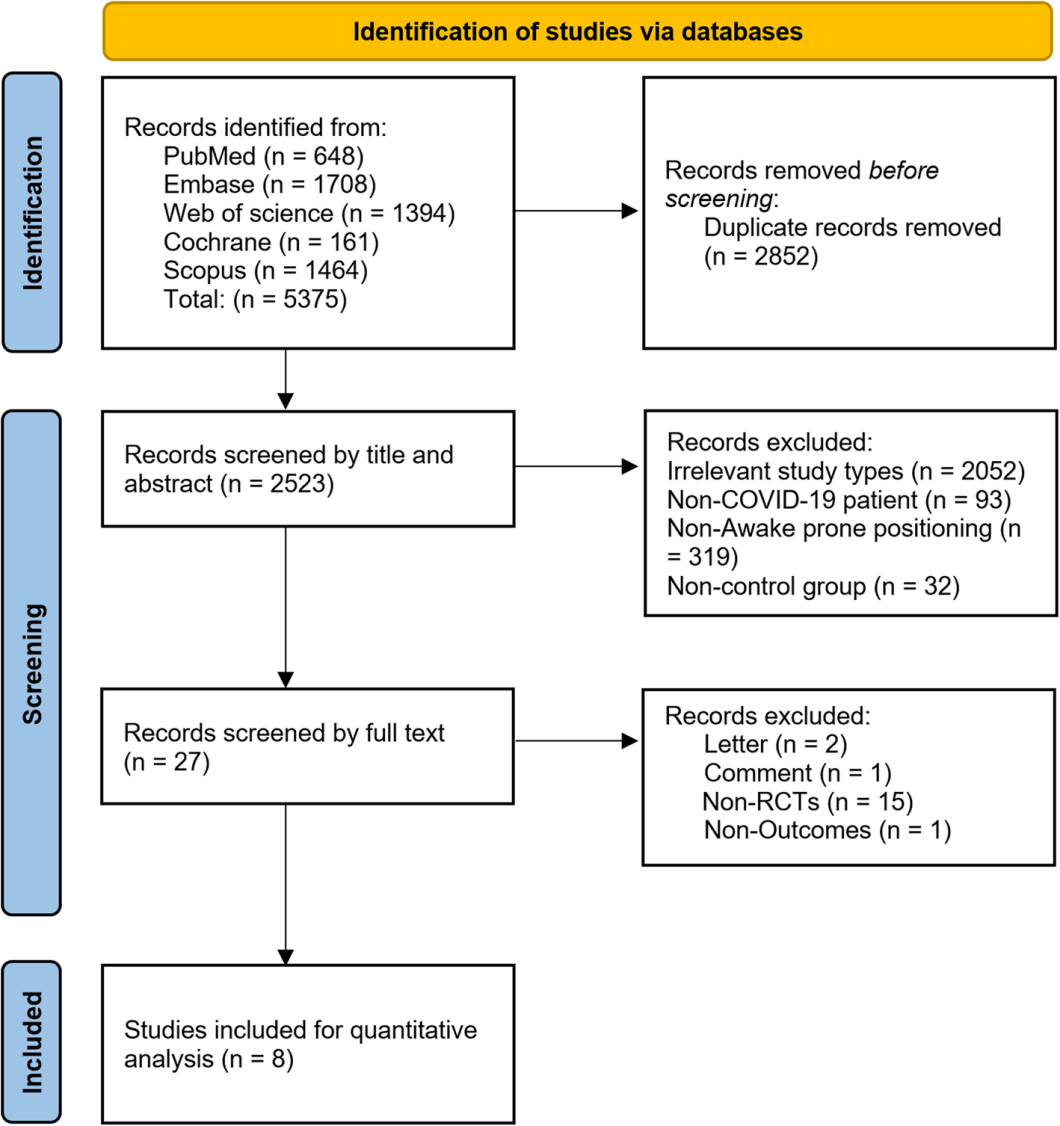
The PRISMA flow chart

We obtained 5,375 articles from five databases, and finally eight RCTs [16, 17, 21–26] met the inclusion criteria, all of which were published between 2021 and 2022 (Fig. 1). A total of 2,657 patients with AHRF caused by COVID-19 were enrolled, including 1,351 in awake prone positioning and 1,306 in usual care. The prone positioning protocols varied in duration and frequency, but all studies encouraged patients to be in the prone positioning whenever possible. The way patients receive oxygen supply included nasal prong, face mask, non-rebreather mask (NRB), high flow nasal cannula (HFNC), non-invasive ventilation (NIV). Research sites included intensive care unit (ICU), general ward, and high-acuity units. The sample size of a single study ranged from 30 [23]-1,121 [25]. Table 1 in the text shows more detailed basic information.

**Table 1:** Basic information of included studies

**Author**	**Year**	**Country**	**Study type**	**Disease**	**Oxygen supply**	**Setting**	**Awake prone positioning**			**Standard care**		
							**Sample size**	**Age**	**Care details**	**Sample size**	**Age**	**Care details**
Jayakumar	2021	India	RCT	COVID‑19‑induced AHRF	Nasal prong, face mask, NRB, HFNC, NIV	ICU	30	54.8 ± 11.1	Awake prone positioning for at least 6 h per day	30	57.3 ± 12.1	Those patients were permitted to change positions as needed for their comfort (supine, semirecumbent, sitting or lateral)
Gad	2021	Egypt	RCT	COVID‑19‑induced AHRF	NRB	ICU	15	49.0 (38–62)	Each session last for 1 to 2 h according to patient to tolerability with 3hs apart during waking hours	15	46.0 (33–51)	Unrestricted body position
Fralick	2022	Canada	RCT	COVID‑19‑induced AHRF	Nasal prong, face mask, HFNC	General ward	126	59.5 (45–68)	Awake prone positioning for four times a day (up to two hours for each session) and encouraged to sleep in awake prone positioning overnight	122	54 (44–62)	Unrestricted body position
Rosén	2021	Sweden	RCT	COVID‑19‑induced AHRF	HFNC or NIV	General ward	36	66 (53–74)	At least 16 h awake prone positioning per day. Prone and semi-prone positioning was allowed	39	65 (55–70)	Unrestricted body position
Ibarra	2022	Mexico	RCT	COVID‑19‑induced AHRF	HFNC	High-acuity units	216	58.6 ± 15.8	Patients in the awake prone positioning group were consistently encouraged by the bedside clinicians to remain in awake prone positioning	214	58.2 ± 15.8	awake prone positioning was discouraged. If awake prone positioning was performed for ≥ 1 h, patients were excluded from the per-protocol analysis
Alhazzani	2022	Canada	RCT	COVID‑19‑induced AHRF	HFNC	ICU	205	56.8 ± 12.5	Awake prone positioning was 8 h/d to 10 h/d with 2 to 3 breaks (1–2 h each)	195	58.3 ± 13.2	Nurses instructed patients not to position themselves in the prone position
Ehrmann	2021	France	RCT	COVID‑19‑induced AHRF	HFNC	ICU/General ward	564	61.5 ± 13.3	Patients were instructed and assisted to lie in the prone position for as long and as frequently as possible each day	557	60.7 ± 14	Patients received standard care with high-flow nasal cannula. The use of Awake prone positioning as intervention was discouraged
Rampon	2022	USA	RCT	COVID‑19‑induced HRF	HFNC	General ward	159	52 (39–62)	Four times daily for 1 to 2 h each session and nightly for a total of 12 h	134	54 (43–63)	Lie in bed in whichever position was comfortable

*AHRF* Acute hypoxemic respiratory failure, *NRB* Non-rebreather mask, *HFNC* High flow nasal cannula, *NIV* Non-invasive ventilation, *ICU* Intensive care unit

### Bias risk assessment results

**Fig. 2:**
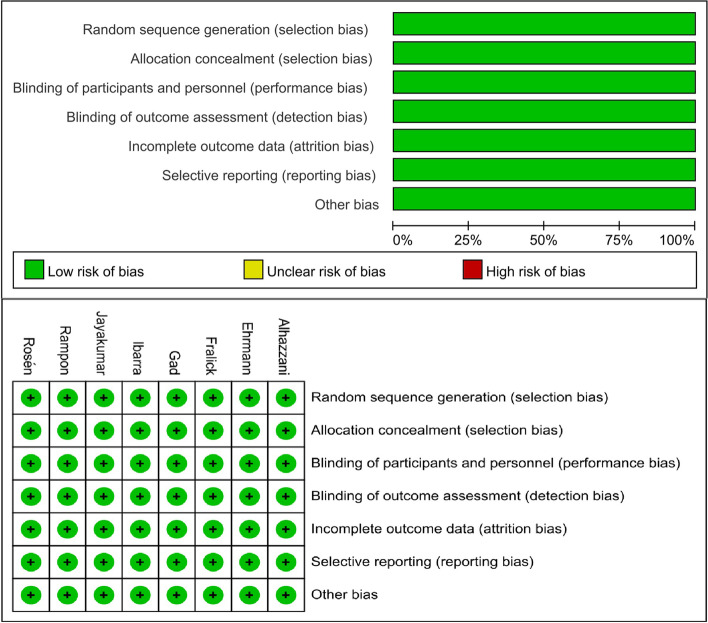
Bias risk assessment results. (A. Risk of bias graph. B. Risk of bias summary)

All eight studies used computerized randomization to group patients. Distributive concealment and blindness were not applicable due to differences in ventilatory posture, but all studies reported the details of the trials. The blind method was applied to the evaluators of the results in all studies. The number of patients who lost follow-up in both the awake prone positioning group and the routine nursing group was less than 10%, and the missing data had similar reasons. Six studies registered protocols and reported their results unselectively. Although the protocols are not available for the other two studies, we do not think that selective reporting exists according to the details of the design, implementation, and reporting of the trials. In conclusion, the included studies all had high quality of evidence. The results of the risk of bias assessment are shown in Fig. 2.

### Meta-analysis results

#### Primary outcomes

**Fig. 3:**
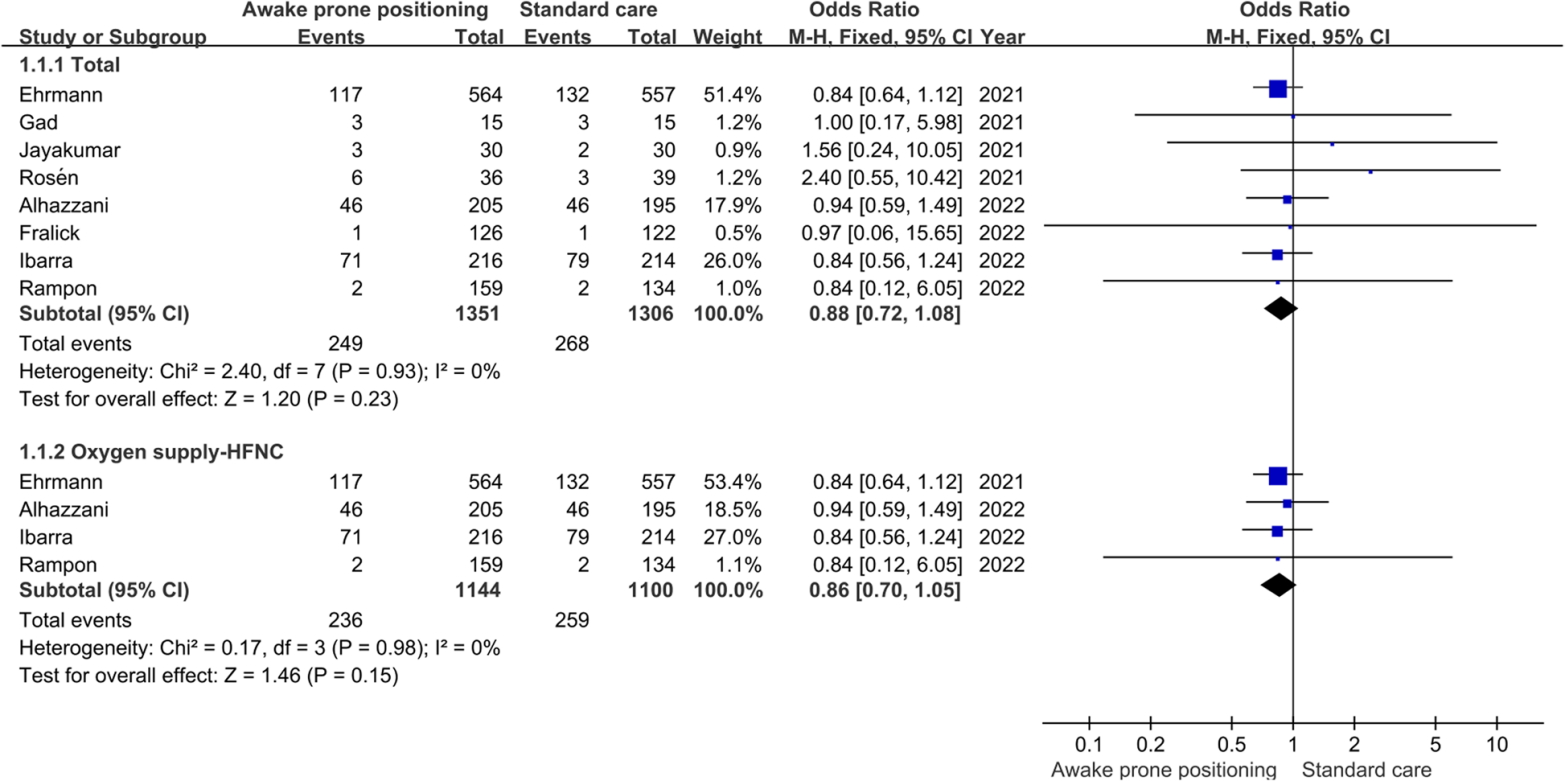
Meta-analysis results of mortality

**Fig. 4:**
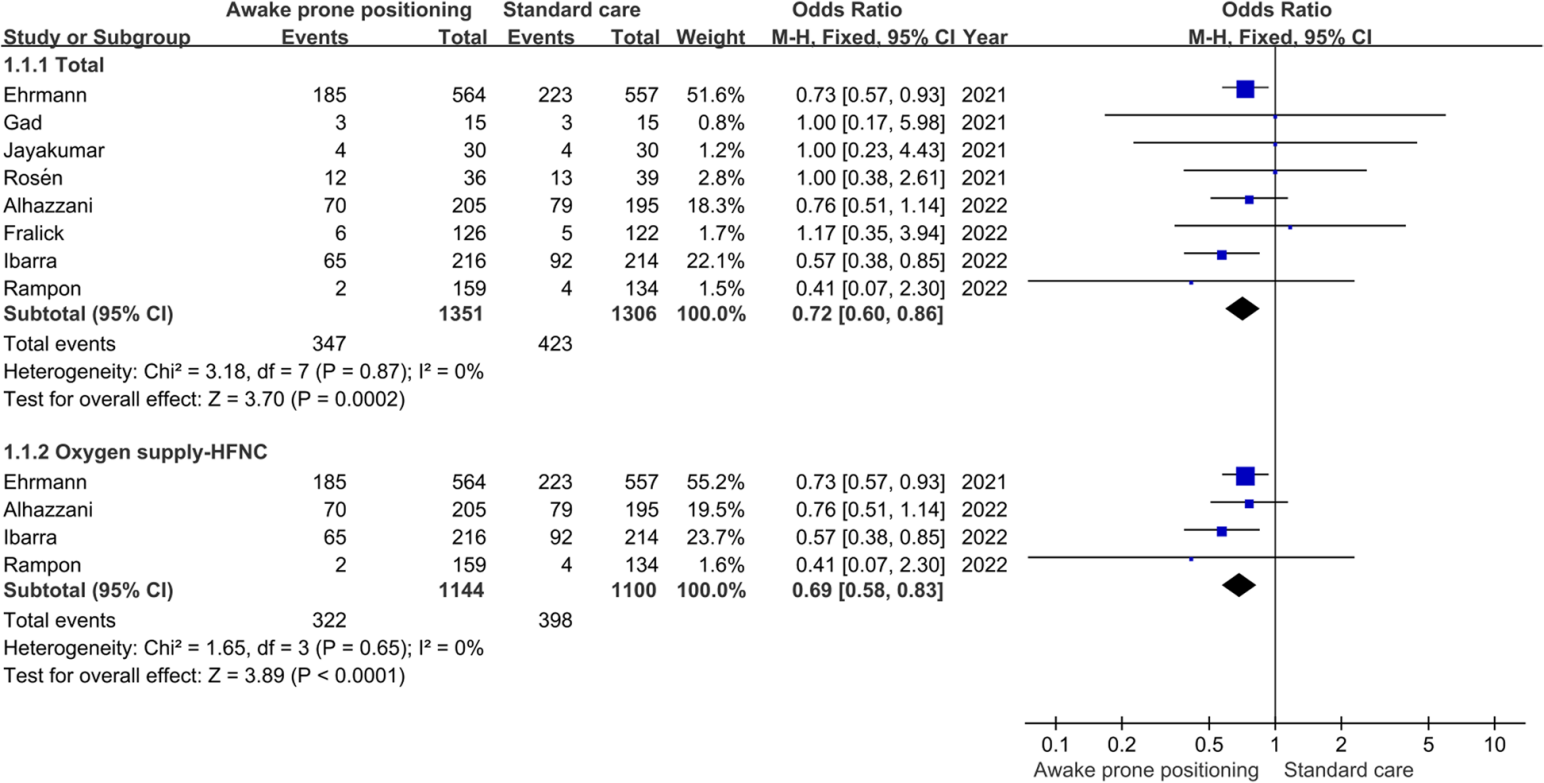
Meta-analysis results of intubation rate

Intubation rate and mortality were reported in all eight studies. The heterogeneity among different studies was very small (I^2^ = 0, P > 0. 05), so the fixed effect model was used. The results of meta-analysis showed that, whether for all patients or only patients who received HFNC, awake prone positioning did not reduce the mortality of patients with AHRF caused by COVID-19 compared with routine care (Total: OR = 0.88, 95%CI [0.72,1.08]. HFNC: OR = 0.86, 95%CI [0.70,1.05] (Fig. 3), but significantly reduced the intubation rate of patients (Total: OR = 0.72, 95%CI [0.60,0.86]; HFNC: OR = 0.69, 95%CI [0.58,0.83]) (Fig. 4).

#### Secondary outcomes

**Fig. 5:**
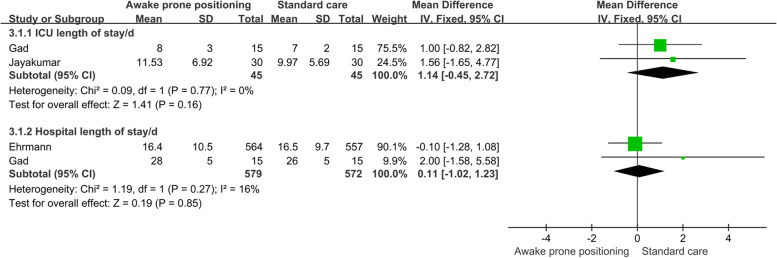
Meta-analysis results of hospitalization time

Two studies reported the length of stay in ICU and the total length of stay in patients. The results of meta-analysis based on the fixed effect model showed that there was no significant difference in ICU hospital stay (WMD = 1.14, 95%CI [-0.45, 2.72]) and total hospital stay (WMD = 0.11, 95%CI [-1.02, 1.23]) between awake prone positioning group and usual care group (Fig. 5).

**Fig. 6:**
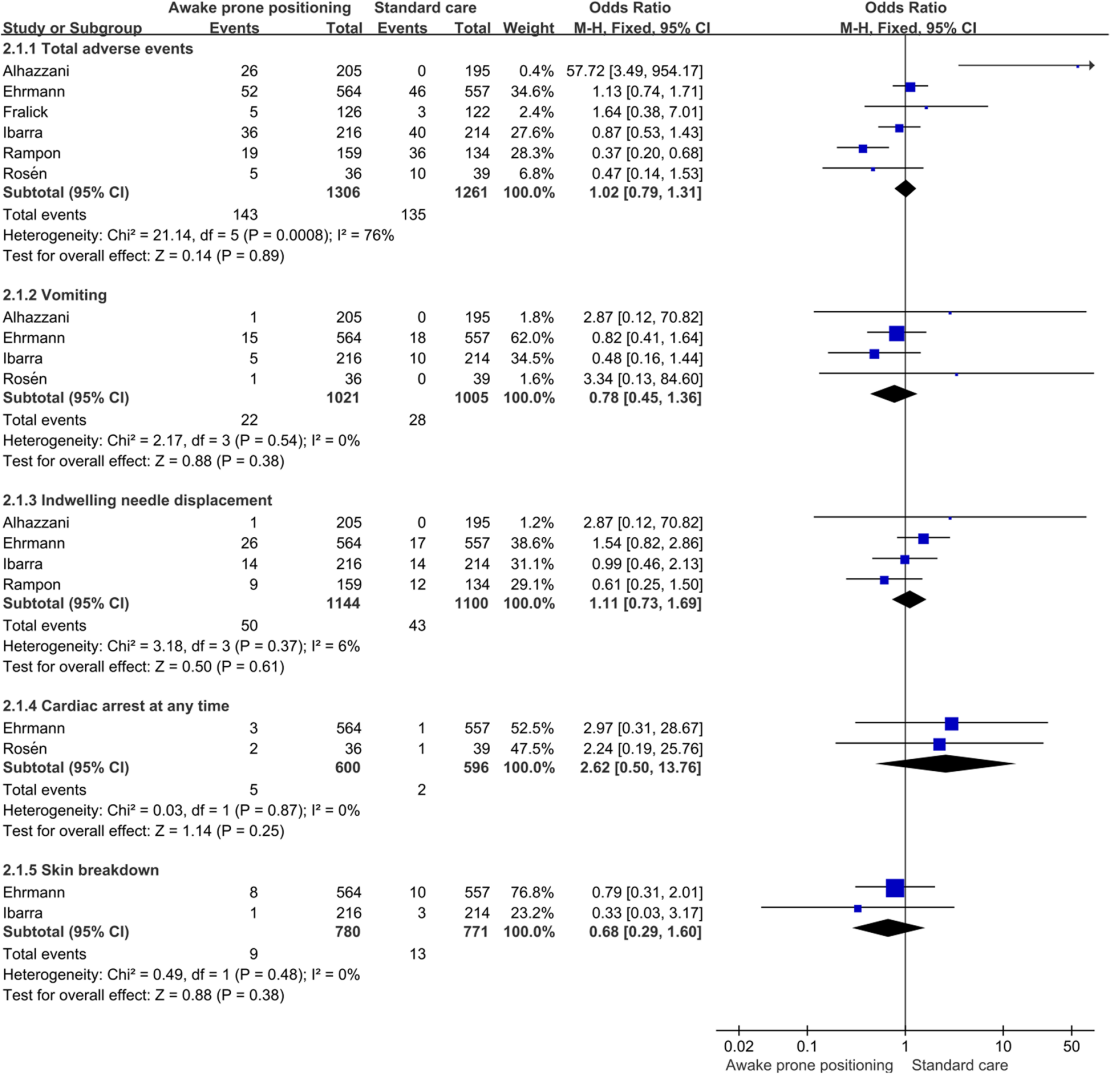
Meta-analysis results of adverse events

A total of six studies reported adverse events of 13 types of patients during treatment. The results of meta-analysis of fixed effect model showed that there was no significant difference in the incidence of total adverse reactions, vomiting, indwelling needle displacement, cardiac arrest at any time and skin breakdown between awake prone positioning group and usual care group (Fig. 6). In addition, the other 9 adverse reactions were reported in only one study, so there was no aggregate analysis (Table 2).

**Table 2:** Incidence of adverse events

**Author**	**Year**	**Adverse events**	**Awake prone positioning**		**Standard care**	
			**Incidence**	**Total**	**Incidence**	**Total**
Fralick	2022	Aspiration pneumonia	2	126	1	122
Fralick	2022	Venous thromboembolism	3	126	2	122
Rosén	2021	Pressure sores	2	36	9	39
Ibarra	2022	Back pain	16	216	13	214
Alhazzani	2022	Hypotension	1	205	0	195
Alhazzani	2022	Shortness of breath	1	205	0	195
Alhazzani	2022	Dizziness	1	205	0	195
Alhazzani	2022	Coughing	1	205	0	195
Rampon	2022	Loss of a urinary catheter	1	159	0	134

#### Publication bias

Publication bias was measured quantitatively by egger's test. The results showed that the *P* value was 0.182, which indicated that there was no publication bias.

## Discussion

The use of awake prone positioning can be traced back to 1977. Douglas et al. [27] performed face mask oxygen inhalation combined with awake prone positioning on respiratory failure patients with pancreatitis and pulmonary edema. The blood oxygen saturation was significantly improved and tracheal intubation was avoided. Scaravilli et al. [28] used the awake prone positioning to treat non-intubated patients with hypoxic respiratory failure, resulting in significantly better therapeutic outcomes than the supine position. Ding et al. [7] found that the intubation rate of moderate ARDS patients could be reduced to 33% by using awake prone positioning combined with high flow nasal catheter oxygen inhalation and non-invasive ventilation device. The successful practice of awake prone positioning in patients with hypoxic respiratory failure caused by other diseases suggests that it may be a promising treatment for patients with hypoxic respiratory failure caused by COVID-19. However, supporting evidence is limited to case reports, cohort studies, and low-quality RCTs. Although these studies are important sources of evidence to guide clinical practice, they lack key trial details and scientific methods to ensure reliable research results. In addition, some studies have reported the opposite results. The results of the randomized controlled trial (RCT) by Johnson et al. [10] showed that awake prone positioning did not reduce the intubation rate and mortality in patients compared to usual care, and they concluded that awake prone positioning was not feasible in these patients. A pragmatic non-RCTs study conducted by Qian et al. [11] to evaluate the benefits of intervention under routine real-life operating conditions also found that awake prone positioning did not reduce intubation rates, length of stay, or 28-day mortality in patients with COVID-19 induced AHRF. In conclusion, the use of awake prone positioning in the treatment of patients with AHRF caused by COVID-19 remains controversial.

Previous meta-analysis of cohort studies and RCTs showed that awake prone positioning significantly reduced mortality compared with usual care, but did not reduce intubation rate [29]. The results of meta-analysis by Kang et al. showed that awake prone positioning could reduce the mortality of COVID-19-induced AHRF or ARDS patients. In fact, the data sources of the previously published SRs/MAs are mainly observational studies [12]. The study design may have a potential impact on the outcome of patients. Although observational studies are real-world studies, the design principles of observational studies are mainly non-randomization, non-intervention and openness, which may lead to bias in the implementation of the test and the measurement of the results [30]. And RCTs overcomes the limitations of observational studies. In addition to the type of study design, the quality of evidence in the study also affects the reliability of the meta-analysis results. The previously published meta-analysis found that the included study had a high risk of bias [12], or the quality of the evidence included in the study was limited [15]. Therefore, these studies recommend that future data from RCTs are needed to further explore the effects of awake prone positioning on patients with AHRF caused by COVID-19. It is worth noting that our meta-analysis is based on high-quality RCTs data, so the results are more reliable. We found that although awake prone positioning did not reduce the mortality of patients, it could significantly reduce the intubation rate in patients. The possible reasons include that the ventilation distribution in prone positioning is more uniform than that in supine position, which can reduce alveolar shunt and achieve an appropriate ventilation blood flow ratio [31, 32]. In addition, when the patient is in the prone positioning, affected by gravity, it is more conducive to the clearance of dorsal lung secretions [33]. At the same time, the collapsed alveoli on the dorsal side of the lung tend to reopen [34]. Obviously, even though the results of our meta-analysis differed from or even reversed the results of previously published meta-analyses, the data based on high-quality RCTs ensured the reliability of our findings.

In fact, among the eight high-quality RCTs included in our meta-analysis, there were differences in the results of different studies. For example, in an analysis based on 60 patients with AHRF due to COVID-19, Jayakumar et al. found no statistically significant differences in any outcomes such as intubation rate and mortality between patients who received awake prone positioning and usual care [22]. However, as the authors claim, the implementation of awake prone positioning is challenging, and only 43% of patients can follow the treatment plan of prone positioning for at least 6 h a day. The authors also believe that the study is a feasibility study and the findings cannot change clinical practice. At the same time, the study was affected by the small sample size. Recently, a large RCT study based on 430 patients by Ibarra et al. provided stronger evidence [16]. They found that awake prone positioning could significantly reduce the intubation rate of patients and improve the success rate of treatment. In short, in view of the baseline characteristics of patients in different studies, such as age, sex, body mass index, disease severity, complications, as well as differences in the implementation plan and acceptance of awake prone positioning, the differences between the results of different studies are predictable and acceptable [35, 36]. Based on this difference, our meta-analysis ensures the homogeneity of the studies as much as possible by establishing strict inclusion and exclusion criteria. The I^2^ of heterogeneity test is zero, and *P* > 0.05 proves this point. Therefore, the current conclusive evidence suggests that awake prone positioning can significantly reduce the risk of endotracheal intubation in patients with AHRF caused by COVID-19 without increasing mortality.

Another interesting aspect of awake prone positioning is safety. Possible complications in prone positioning include indwelling needle displacement, transient hypotension, vomiting and pressure sores [34]. Our results showed that awake prone positioning did not increase the incidence of adverse events compared with usual care. Although the studies by Rosén et al. [37] and Ehrmann et al. [38] reported serious adverse events of cardiac arrest, the authors claimed that this was not related to prone positioning. In addition, we analyzed the length of stay of patients receiving different care modes and found that awake prone positioning did not increase patients' time in ICU or hospital stay. In short, awake prone positioning is safe and feasible for patients with AHRF caused by COVID-19.

Recent studies suggest that longer durations of prone positioning are associated with better patient outcomes [16, 19, 38]. The study of Carsetti et al. showed that prolonging the duration of prone positioning (average 36 h) could better improve oxygenation and maintain the improved oxygenation when restoring posture compared with patients in prone positioning for 16 h [39]. Eperatti et al. recommended at least 8 h of prone positioning per day to reduce the risk of death [19]. However, since the patient remained awake, he could not tolerate continuous prolonged awake prone positioning (12–16 h per day) [6, 40]. Most studies have found that the average time for patients to tolerate prone positioning is 2 to 3 h each time [41–43]. In short, the prone positioning should be extended as long as the patient can tolerate it. The cumulative time of prone positioning can be increased by taking prone positioning many times a day, and the tolerance of patients can be improved through the transformation of prone positioning, right supine position, high sitting position and left supine position [44, 45]. In addition, mild sedation can also improve patient tolerance, but requires close monitoring of patient respiratory status [46]. It is worth noting that although the duration of ventilation in the prone positioning will affect the effectiveness of treatment, the current RCTs does not explore the actual effect of the duration of ventilation in the prone positioning on patients. This should be the focus of future research. Also, awake prone positioning is a complex medical intervention, and there are many nuances in the implementation protocols of different studies, such as adoption rates, feasibility, and tolerability, that may affect the successful implementation of RCTs and the reliability of outcomes. In short, due to the lack of standardized procedures, the strategy of awake prone positioning is not consistent. The optimal frequency, duration, and criteria for starting or stopping prone positioning are unclear. Although no significant adverse events attributable to awake prone positioning have been reported, awake prone positioning is not without limitations and has been associated with intolerance, discomfort, and anxiety [15]. Therefore, it is necessary to further explore the implementation strategy of awake prone positioning in the future, so as to provide a scientific guidance for clinical practice.

## Limitations

As the first meta-analysis of RCTs data in the current field, the quality of the studies we included is very high, and the statistical heterogeneity between different studies is within an acceptable range, which ensures the reliability of meta-analysis results to a great extent. Although statistical heterogeneity is acceptable, the impact of clinical heterogeneity and methodological heterogeneity on meta-analysis results cannot be estimated. For example, the source of patients includes ICU, general ward, and high-acuity units, and the severity of disease varies among patients in different locations. The amount of time patients were given prone ventilation also varied considerably across studies (Prone positioning for at least 6 h [22], 16 h [37], or encouraged to stay in prone positioning all the time[16]). In addition, blinding of trial implementers and patients was unrealistic in the included studies, but we judged that failure to implement blinding did not affect the effect of the intervention based on the implementation details of the studies. This practice is likely to exaggerate the quality of studies. Also, considering the credibility of the results, we include only published studies, not grey studies that have not been peer reviewed, so we may ignore some important findings. In addition, due to the unavailability of data, it is not possible to estimate the impact of adjuvant therapy on the effectiveness of interventions.

## Conclusions

The latest evidence from high-quality RCTs suggests that awake prone positioning is safe and feasible for non-intubated patients with AHRF caused by COVID-19 and does not lead to more adverse events than usual care. Awake prone positioning can significantly reduce the intubation rate without increasing the mortality. However, the implementation strategy of awake prone positioning still needs more research.

### Supplementary Information

**Additional file 1:** **Table 1.** [Search strategies].

## Data Availability

All data generated or analyzed during this study are included in this published article.

## Abbreviations

AHRF: Acute hypoxic respiratory failure
RCTs: Randomized controlled trials
ICU: Intensive care unit
ARDS: Acute respiratory distress syndrome
SRs/MAs: Systematic reviews/Meta-analyses
PRISMA: Preferred Reporting Items for Systematic Reviews and Meta-Analyses
OR: Odds ratio
WMD: Weighted mean difference
NRB: Non-rebreather mask
HFNC: High flow nasal cannula
NIV: Non-invasive ventilation
