# Supplementary material for: Awake prone positioning for non-intubated patients with COVID-19-related acute hypoxic respiratory failure: a systematic review based on eight high-quality randomized controlled trials
Source: BMC Infect Dis. 2023 Jun 19;23:415. doi: 10.1186/s12879-023-08393-8 (PMC10278266; doi:10.1186/s12879-023-08393-8)
Supplement: Supplementary file 1 — Additional file 1: Table 1. [Search strategies]. [file 12879_2023_8393_MOESM1_ESM.docx]

**Awake prone positioning for non-intubated patients with COVID-19-related acute hypoxic respiratory failure: A systematic review based on eight high-quality randomized controlled trials**

**Table 1: Search strategies**

| **1. WOS**  (TS=(supine position OR dorsal position OR prone position OR lateral position OR ventilation position OR ventilatory position OR ventilation posture OR ventilatory posture)) AND TS=(SARS-CoV-2 OR SARS-CoV2 OR ‘severe acute respiratory syndrome coronavirus 2’ OR 2019-nCoV OR 2019nCoV OR coronavirus OR covid-19 OR COVID19 OR COVID-19) 1394  **2. PubMed**  #1: "Supine Position"[MeSH Major Topic] OR "Prone Position"[MeSH Major Topic] 2685  #2: supine position[Title/Abstract] OR dorsal position[Title/Abstract] OR prone position[Title/Abstract] OR lateral position[Title/Abstract] OR ventilation position[Title/Abstract] OR ventilatory position[Title/Abstract] OR ventilation posture[Title/Abstract] OR ventilatory posture[Title/Abstract] 24279  #3: #1 OR #2 25474  #4: "SARS-CoV-2"[Title/Abstract] OR "SARS-CoV2"[Title/Abstract] OR "severe acute respiratory syndrome coronavirus 2"[Title/Abstract] OR "2019-nCoV"[Title/Abstract] OR "2019nCoV"[Title/Abstract] OR "coronavirus"[Title/Abstract] OR "covid 19"[Title/Abstract] OR "COVID19"[Title/Abstract] OR "covid 19"[Title/Abstract] 324837  #5: "SARS-CoV-2"[MeSH Terms] OR "Coronavirus"[MeSH Terms] OR "COVID-19"[MeSH Terms] 217907  #6: #4 OR #5 338164  #7: #3 AND #6 648  **3. Cochrane**  #1: MeSH descriptor: [Supine Position] explode all trees 685  #2: MeSH descriptor: [Prone Position] explode all trees 334  #3: ("supine position" OR "dorsal position" OR "prone position" OR "lateral position" OR "ventilation position" OR "ventilatory position" OR "ventilation posture" OR "ventilatory posture"):ti,ab,kw 7157  #3: #1 or #2 or #3 7157  #4: (SARS-CoV-2 OR SARS-CoV2 OR ‘severe acute respiratory syndrome coronavirus 2’ OR 2019nCoV OR coronavirus OR covid-19 OR COVID19 OR COVID-19):ti,ab,kw 14305  #5: MeSH descriptor: [SARS-CoV-2] explode all trees 1187  #6: MeSH descriptor: [COVID-19] explode all trees 2553  #7: MeSH descriptor: [Coronavirus] explode all trees 1204  #8: #5 or #6 or #7 or #8 14311  #9: #3 and #8 161  **4. Embase**  #1: 'supine position':ab,ti OR 'dorsal position':ab,ti OR 'prone position':ab,ti OR 'lateral position':ab,ti OR 'ventilation position':ab,ti OR 'ventilatory position':ab,ti OR 'ventilation posture':ab,ti OR 'ventilatory posture':ab,ti 31982  #2: 'supine position'/exp 26333  #3: 'dorsal position'/exp 26333  #4: 'prone position'/exp 6337  #5: 'lateral position'/exp 120  #6: #1 OR #2 OR #3 OR #4 OR #5 47230  #5: ('sars cov 2':ab,ti OR 'sars cov2':ab,ti OR '2019 ncov':ab,ti OR 'severe acute respiratory syndrome coronavirus 2':ab,ti OR 2019ncov:ab,ti OR coronavirus:ab,ti OR covid19:ab,ti OR 'covid 19':ab,ti) AND [2019-2022]/py 337824  #6: 'sars cov 2'/exp 82740  #7: 'coronavirus'/exp 112959  #8: 'covid 19'/exp 281390  #9: #6 OR #7 OR #8 387668  #19: #5 AND #10 1708  **5. Scopus**  (TITLE-ABS-KEY("supine position" OR "dorsal position" OR "prone position" OR "lateral position" OR "ventilation position" OR "ventilatory position" OR "ventilation posture" OR "ventilatory posture") AND TITLE-ABS-KEY(SARS-CoV-2 OR SARS-CoV2 OR "severe acute respiratory syndrome coronavirus 2" OR 2019-nCoV OR 2019nCoV OR coronavirus OR covid-19 OR COVID19 OR COVID-19)) 1464 |
| --- |
